# Personality-oriented job analysis to identify non-cognitive factors predictive of performance in a doctor of physical therapy program in the United States

**DOI:** 10.3352/jeehp.2018.15.34

**Published:** 2018-12-28

**Authors:** Maureen Conard, Kristin Schweizer

**Affiliations:** 1 Department of Psychology, Sacred Heart University, Fairfield, CT, USA; 2 Department of Physical Therapy, Sacred Heart University, Fairfield, CT, USA; Hallym University, Korea

**Keywords:** Personality, Non-cognitive factors, Doctorate of physical therapy students, Surveys and questionnaires, United States

## Abstract

This study aimed to conduct a personality-oriented job analysis to identify non-cognitive factors that may predict successful performance or performance difficulties in doctor of physical therapy (DPT) students. The study employed focus groups and a survey with 9 DPT subject matter experts. The focus group participants, who included 3 DPT faculty members and 4 recent graduates of the DPT program, identified 22 non-cognitive factors. Fifteen of these factors were thought to be possibly associated with successful performance and 7 factors were thought to be possibly associated with performance difficulties. Administration of a questionnaire employing the combination job analysis method resulted in 12 factors that could be used in selection, and 10 that could be incorporated into training. The present study employed an established job analysis method using subject matter experts to identify a broad array of factors that go beyond what previous studies have examined, and which may predict success or difficulties in a DPT program.

Successful completion of the doctor of physical therapy (DPT) education program and the National Physical Therapy Examination (NPTE) are key outcome measures for physical therapist education programs. Minimum expectations for these outcomes are established by the Commission on Accreditation in Physical Therapy Education and institutional performance on these outcomes contribute to institutional reputation.

One goal of the admissions process is to select applicants who have a strong likelihood of success in the DPT education program and on the NPTE. Currently, DPT education programs use a combination of cognitive and non-cognitive factors to select appropriate candidates for their programs. Cognitive factors used in admissions decisions may include undergraduate grade point average (GPA), prerequisite course GPA, and Graduate Record Examination (GRE) scores. Measures of non-cognitive factors are frequently obtained through essays, letters of recommendation, and interviews.

Predictors of student success in DPT education programs and on the NPTE have been the focus of numerous studies. Utzman et al. [1,2] found that demographic factors such as age and race/ethnicity and quantitative cognitive data including GPA and GRE scores account for 24% and 28% of the variability in academic and NPTE success, respectively. The evidence regarding measures of non-cognitive factors evaluated through essays, letters of recommendation, observation hours and interviews to predict academic and NPTE success is conflicting [3-8].

Increased interest has emerged in personality factors that influence student performance. Kappe and van der Flier [9] found that 33% of the variance in GPA and 30% of the variance in time to graduation in higher education could be attributed to a combination of student-related factors measured by the Big Five Personality Inventory. The Big Five Personality Inventory [10] and the Non-Cognitive Questionnaire-Revised [11] did not predict scores on the NPTE in studies limited to a single physical therapy education program. In graduate nursing students, a strong correlation was found between resilience and academic success [12]; however, this relationship has not yet been studied in DPT education. Roll et al. [13] developed a survey measuring non-cognitive factors, such as emotional intelligence and grit, that was intended for use in DPT program admissions processes. This survey’s ability to predict students’ success has yet to be validated.

Personality-oriented job analysis (POJA) is a widely accepted method used by industrial and organizational psychologists to identify personality factors that may contribute to success in a job role. In a study conducted to determine whether those personality factors impacted medical school students’ academic and clinical performance, it was found that personality factors accounted for 8.2% and 7.8% more variance in academic and clinical performance, respectively, than the traditionally used measures alone [14].

The present study is a POJA for DPT students. It presents the first 2 phases (focus group and POJA questionnaire) of a planned validation study to investigate the extent to which non-cognitive factors can predict academic performance, retention, clinical performance, and performance on the NPTE. The POJA process is described and results are presented. The results will be used to determine which non-cognitive factors will be used in the validation phases of the study.

## Ethical statement

This study was approved by the Institutional Review Board of Sacred Heart University (IRB approval no., 180130A). Informed consent was obtained from the participants.

## Study design

The first phase of the study consisted of focus groups with DPT subject matter experts (SMEs). The second phase was a cross-sectional survey of SMEs from 1 university.

## Materials and subjects

### Phase I: focus groups

*Participants:* SMEs were identified by 1 of the authors and were invited to participate. Three DPT faculty members (all classroom instructors, 1 of whom also oversaw clinical rotations) participated in one group. Four recent graduates of the DPT program participated in a separate group by conference call.

*Materials and procedures:* A list of 34 possible POJA traits were identified from the department’s list of essential functions for physical therapists [15], as well as from previous healthcare-related studies [8,14]. Descriptions of items were obtained from the International Personality Item Pool website and were edited for clarity. Research in leader performance has increasingly shown the importance of studying both negative and positive factors that affect performance, as they can coexist in people. Negative factors can derail what would otherwise be positive performance [16,17,18]. Therefore, the stimulus list purposely included factors that could be detrimental to performance or lead to performance difficulties.

SMEs were given the stimulus list of POJA traits and definitions and they were asked to critique them for clarity, to condense and combine traits and definitions, and to add any new traits if appropriate. This resulted in a list of 22 traits and definitions, 15 of which could be associated with successful performance, and 7 which could be associated with performance difficulties.

### Phase II: personality-oriented job analysis questionnaire

*Participants:* Eleven SMEs were recruited to participate in the study. Nine SMEs participated, including 6 DPT faculty members and 3 recent graduates who had passed the NPTE and were employed as physical therapists.

*Materials and procedure:* A questionnaire with 22 POJA traits and definitions was developed and is presented in the Appendix 1. The response scales for the personality factors were adapted from the knowledge, skills, abilities, and other characteristics sections of Levine’s combination job analysis method (C-JAM) as described by Brannick et al. [19]. The wording of the scales was modified to be appropriate for student admissions rather than job applicants. The SMEs rated traits on 4 scales: (1) Is this factor necessary for new DPT students (yes/no)?; (2) Is this factor practical to expect among DPT applicants (yes/no)?; (3) To what extent is trouble likely if ignored in admissions? (rated on a 5-point scale: 1= very little or none to 5= to an extremely great extent); and (4) To what extent do different levels of the factor distinguish the superior from average student (rated on 5-point scale, 1= very little or none to 5= to an extremely great extent).

For the 7 factors that were thought to be possibly associated with performance difficulties, the first 2 scales were reworded to “Is the factor detrimental for new first-year DPT students?” and “Is the factor common among DPT applicants?”

Previous POJA studies identified several response errors, including self-serving bias, implicit trait policies, social projection, and false consensus. A randomized trial of Aguinis et al. [20] showed that frameof-reference (FOR) training reduced bias, decreased the correlation between SMEs’ own personality traits and the POJA trait ratings, and decreased overall POJA ratings, compared to a group given standard instructions. A subsequent meta-analysis showed that FOR training improved the accuracy of ratings [21]. Therefore, specific instructions were provided to reduce response errors and to improve accuracy. SMEs were presented with the following instructions at the beginning of the questionnaire: “When considering the factors it is important to think about DPT students in general, not just yourself as you were as a student. For example, while you may have always kept your desk area very neat and as organized as possible, many students may not do this and they may still be successful.” The questionnaire was created in SurveyMonkey (http://www.surveymonkey.com) and a link was emailed to 11 SMEs. The raw data are available in Supplement 1.

## Technical information

The C-JAM process involves computing the number of SMEs who answered “yes” to (1) whether the trait is necessary for new students and (2) whether the trait is practical to expect among applicants [10]. Additionally, mean scores were computed for (1) trouble being likely if a trait is ignored in admissions, and (2) whether the trait would distinguish superior from average students. If a majority of SMEs answer that the trait is necessary and practical to expect and the mean score for trouble being likely if a trait is ignored is greater than 1.5, then the trait can be incorporated into selection procedures. For the traits to be used in selection, if the mean score for its ability to distinguish superior from average students is greater than 1.5, then selection procedures including it should result in a ranked ordering of applicants. If the mean score for distinguishing superior from average students is equal to or lower than 1.5, a pass/fail selection procedure should be developed.

C-JAM also has decision rules for which traits can be used in training. If less than a majority of SMEs agree that the trait is necessary for new students and the mean score for its ability to distinguish superior from average students is greater than 1.5, it is recommended for training.

The present study included several methodological improvements. First, 5-point rating scales were used, rather than 3-point scales, to allow for more nuanced variation in ratings [22]. Second, SMEs answered 4 questions about each factor, and the structured decision-making process of C-JAM was used to decide which personality factors to include in selection and which to recommend for training. Third, FOR instructions for SMEs were included, which have been shown to decrease self-serving bias, implicit trait policies, social projection, and false consensus, and to improve accuracy [20,21].

The focus group participants, including 3 DPT faculty members and 4 recent graduates of the DPT program, identified 22 non-cognitive factors. Fifteen factors were thought to be possibly associated with successful performance, and 7 factors were thought to be possibly associated with performance difficulties.

Table 1 presents the questionnaire results for factors to be considered for selection. Following the previously outlined decision rules for C-JAM, the SME ratings resulted in 12 factors to be considered for selection (11 desirable factors and 1 undesirable factor). Table 2 presents the results for factors to be considered for training. The SME ratings also yielded 10 factors recommended for training (4 desirable factors and 6 undesirable factors).

The C-JAM process resulted in 12 factors to be included in selection procedure validation, in addition to the cognitive procedures currently in place. Several of these factors aligned with the findings of Roll et al. [13]. Specifically, measures of adaptability, interpersonal skills, and tolerance in the POJA were similar to the measures of adaptability, intuitiveness, and engagement presented by Roll et al. [13], respectively. However, the results of the C-JAM analysis identified several additional factors that could be included in the selection process, 8 of which were expected to contribute positively to performance, and 1 of which was expected to contribute to performance difficulties. Additionally, 4 of the top 6 factors identified by McLarnon et al. [14] (conscientiousness, calm/relaxed, tolerance, and responsibility) were also identified in the present study. Of note, however, is that McLarnon et al. [14] utilized a rating scale that ranged from disastrous to essential, and selected the factors that scored the highest. This effectively eliminated any undesirable factors.

The present study separated ratings for desirable and undesirable factors to result in a spectrum of factors. Interestingly, the majority of SMEs indicated that those 6 undesirable factors are not common among applicants. C-JAM rules suggest that they can be recommended for further training. However, since they are uncommon, further discussion with SMEs is warranted regarding the utility of training for such uncommon factors. The present study included several methodological strengths. First C-JAM is a formal, standardized job analysis process with clear decision rules, and the results can be used for both selection and training. Second, performing job analysis and utilizing SMEs in that process is recommended in the ‘uniform guidelines on employee selection procedures’ to ensure validity and improve legal defensibility [23]. Third, while most previous studies have focused on positive factors, the present study included several factors that may lead to performance difficulties. Fourth, the instructions to SMEs included FOR training, which has been shown to reduce bias and to improve accuracy in ratings [20,21]. Fifth, a subsequent validation study will identify scientific and legally defensible non-cognitive measures that could help improve the selection and retention of first-year students, performance in clinical placements, and first-time pass rates on the NPTE.

A limitation is that the DPT education program studied utilizes a problem-based learning curriculum. This curricular model is used by 1.7% of physical therapy education programs nationally [24]. Therefore the results may not generalize to other DPT education curricula, and would need to be verified in other programs.

In conclusion, the present study is the first step in the process of identifying and validating non-cognitive factors that may contribute to success and difficulties in a DPT education program. Previous studies of non-cognitive factors such as demographics or essays have produced conflicting results. Using SMEs, the present analysis identified a broad array of factors that go beyond those examined in previous studies, and which can be validated. The next step will be to conduct a concurrent validation study by administering personality measures of those factors to current DPT students, and correlating scores on the measures with course grades, clinical rotation performance, and NPTE scores.

## Figures and Tables

**Table 1.** Personality factors for selection

Variable	Mean ± standard deviation^a)^
Desirable factors	
Ethics/integrity/honesty	4.22 ± 1.39
Team player/collaborative	3.89 ± 1.17
Resilience/perseverance/grit/poise	3.78 ± 1.09
Analytical	3.56 ± 1.24
Grit	3.55 ± 1.33
Tolerance	3.44 ± 0.88
Accountable/dependable/conscientiousness	3.22 ± 0.83
Interpersonal skills	3.11 ± 1.45
Adaptability	2.67 ± 1.12
Problem sensitivity	2.67 ± 1.12
Problem solving/ decision making/self-reflection	2.44 ± 1.01
Undesirable factors	
Anxiousness/neuroticism	3.00 ± 1.00

a) Trouble likely if ignored in selection.

**Table 2.** Personality factors for training

Variable	Mean ± standard deviation^a)^
Desirable factors	
Inquisitiveness	3.89 ± 0.93
Assertiveness	3.00 ± 1.32
Social confidence	2.78 ± 1.09
Perfectionism	1.89 ± 0.60
Undesirable factors	
Lack of resiliency	4.5 ± 0.72
Lack of accountability	4.33 ± 0.71
Lack of focus	4.22 ± 0.97
Lack of maturity	4.22 ± 1.39
Dominance/aggressiveness	3.22 ± 1.20

a) Distinguishes superior from average students.

## Supplementary material

Supplement 1. Data files are available from https://doi.org/10.7910/DVN/POGIMX

Supplement 2. Audio recording of the abstract.
