# Supplementary material for: Personality-oriented job analysis to identify non-cognitive factors predictive of performance in a doctor of physical therapy program in the United States
Source: J Educ Eval Health Prof. 2018 Dec 28;15:34. doi: 10.3352/jeehp.2018.15.34 (PMC6380910; doi:10.3352/jeehp.2018.15.34)
Supplement: Supplementary file 2 [file jeehp-15-34-app1.pdf]

Appendix 1. POJA questionnaire with factor definitions

Introductory statement

By completing this questionnaire you will help us identify personality traits and other noncognitive factors that are important for doctor of physical therapy (DPT) student success.

When considering the factors it is important to think about DPT students in general, their strengths and weaknesses, and not specifically yourself as a student. For example, while you may have always kept your desk area very neat and as organized as possible, some students may not do this and they may still be successful. Or there may be factors that you did not have, or did not need to have as a student, but are necessary or desirable for DPT students at this institution.

There are four ratings' scales for the questionnaire. Please read through them carefully to familiarize yourself with them. For the last few factors, the first two rating scales are reworded. Please familiarize yourself with the new wording at that point in the questionnaire.

A) Is the factor necessary for new first year DPT students? (Yes / No)

B) Is the factor practical to expect among DPT applicants? (Yes / No)

C) To what extent is *trouble* likely if this factor is ignored in admissions?
1 = Very little or none
2 = To some extent
3 = To a great extent
4 = To a very great extent
5 = To an extremely great extent

D) To what extent do different levels of the factor distinguish the *superior* from the *average* student?
1 = Very little or none
2 = To some extent
3 = To a great extent
4 = To a very great extent
5 = To an extremely great extent

Accountable/dependable/achievement oriented/conscientiousness
A person high on this factor accepts responsibilities, tries to follow rules, follows through with plans, sets high standards for self and others, and is always prepared.

Self-reflection/self-insight/problem solving/decision making
A person high on this factor examines him/herself objectively and assesses his/her own performance to make improvements

Adaptable/flexible
A person high on this factor adjusts easily, responds positively to criticism, and is comfortable with contradictory opinions.

Anxiousness/mental health/neuroticism
A person high on this factor worries often, fears the worst and gets stressed out easily

Perfectionism
A person high on this factor wants every detail taken care of and wants everything to be just right

Resilience/perseverance/grit/poise
A person high on this factor remains calm under pressure and remains steadfast when the going gets tough.

Assertiveness
A person high on this factor easily pushes self, tries to lead others and turns plans into action

Inquisitive/creative/insightful/openness to experience
A person high on this factor asks questions no one else does, challenges others' points of view and can easily link facts together.

Analytical ability
A person high on this factor likes to solve complex problems, tends to analyze things and wants to know the reason why.

Ethics/integrity/honesty
A person high on this factor has good values/morality, keeps promises, and knows honesty is the basis for trust.

Interpersonal skills/empathy/friendliness
A person high on this factor is sensitive to other's thoughts and feelings, warms up quickly to others, takes time out for others, and makes others feel at ease.

Problem sensitivity
A person high on this factor recognizes that there is a problem and can tell when something is wrong or likely to go wrong.

Social confidence/extraversion
A person high on this factor expresses themselves easily and feels comfortable around people.

Team player/collaborative
A person high on this factor enjoys being part of a group, doesn't miss group meetings and respects decisions made by the group.

Tolerance
A person high on this factor respects others, believes there are many sides to most issues and accepts people as they are.

Grit
A person high on this factor is a hard worker, overcomes setbacks to conquer an important challenge, and never gives up.

For the next 6 factors, the first 2 rating scales are reworded. Please familiarize yourself with the new wording before proceeding with the questionnaire.

A) Is the factor *detrimental* for new first year DPT students? (Yes / No)

B) Is the factor common among DPT applicants? (Yes / No)

C) To what extent is *trouble* likely if this factor is ignored in admissions?
1 = Very little or none
2 = To some extent
3 = To a great extent
4 = To a very great extent
5 = To an extremely great extent

D) To what extent do different levels of the factor distinguish the *superior* from the *average* student?
1 = Very little or none
2 = To some extent
3 = To a great extent
4 = To a very great extent
5 = To an extremely great extent

Dominance/domineering/aggressiveness
A person high on this factor bosses people around, insists others do things his/her way, and has a strong need for power and is controlling.

Lack of resiliency
A person high on this factor gives up easily and gets dejected if things aren't going well.

Lack of maturity
A person high on this factor is usually late, often unprepared and irresponsible.

Lack of focus
A person high on this factor doesn't commit enough time to their work and has poor study skills.

Lack of accountability
A person high on this factor doesn't fulfill duties and doesn't use feedback to make improvements.

Lack of responsibility/dependent
A person high on this factor is overly dependent on others for help and support, doesn't meet deadlines and often doesn't follow rules.
